# Nitrogen fertilization form and energetic status as target points conditioning rice responsiveness to elevated [CO_2_]

**DOI:** 10.3389/fpls.2025.1517360

**Published:** 2025-03-11

**Authors:** Ivan Jauregui, Toshiaki Mitsui, Bertrand Gakière, Caroline Mauve, Françoise Gilard, Iker Aranjuelo, Marouane Baslam

**Affiliations:** ^1^ Department of Sciences, Public University of Navarra (UPNA), Pamplona, Spain; ^2^ Laboratory of Biochemistry, Institute for Social Innovation and Cooperation, Niigata University, Niigata, Japan; ^3^ Plateforme Métabolisme-Métabolome, Institut de Biologie des Plantes, Université Paris-Sud, Paris, France; ^4^ AgroBiotechnology Institute (IdAB), Centro Superior de Investigaciones Científicas (CSIC)-Government of Navarre, Mutilva, Spain; ^5^ Centre d’Agrobiotechnologie et Bioingénierie, Unité de Recherche labellisée CNRST (Centre AgroBiotech-URL-CNRST-05), Université Cadi Ayyad, Marrakech, Morocco; ^6^ Laboratory of Agro-Food, Biotechnologies and Valorization of Plant Bioresources (AGROBIOVAL), Department of Biology, Faculty of Science Semlalia, Cadi Ayyad University (UCA), Marrakesh, Morocco; ^7^ GrowSmart, Amsterdam, Netherlands

**Keywords:** rice, low light intensity, elevated CO_2_, nitrogen source, nitrate, plasticity, ATP, photosynthesis

## Abstract

The nitrogen (N) fertilization form and plant energy status are known to significantly influence plant responses to elevated atmospheric carbon dioxide (CO_2_) concentrations. However, a close examination of the interplay between N sources under contrasting light intensity has been notably absent in the literature. In this study, we conducted a factorial experiment with rice plants involving two different light intensities (150 and 300 µmol m^-2^ s^-1^), inorganic N sources [nitrate (N-NO_3_) or ammonium nitrate (N-NH_4_NO_3_)] at varying CO_2_ levels (410 and 700 parts per million, ppm). The aim was to examine the individual and combined effects of these factors on the allocation of biomass in whole plants, as well as on leaf-level photosynthetic characteristics, chloroplast morphology and development, ATP content, ionomics, metabolomics, and hormone profiles. Our research hypothesis posits that mixed nutrition enhances plant responsiveness to elevated CO_2_ (eCO_2_) at both light levels compared to sole N-NO_3_ nutrition, due to its diminished energy demands for plant assimilation. Our findings indicate that N-NO_3_ nutrition does not promote the growth of rice, its photosynthetic capacity, or N content when exposed to ambient CO_2_ (aCO_2_), and is significantly reduced in low light (LL) conditions. Rice plants with N-NH_4_NO_3_ exhibited a higher carboxylation capacity, which resulted in larger biomass (total C, tiller number, and lower root-shoot ratio) supported by higher Calvin-cycle-related sugars. The lower leaf N content and overall amino acid levels at eCO_2_, particularly pronounced in N-NO_3_, combined with the lower ATP content (lowest at LL and N-NO_3_), may reflect the higher energy costs of N assimilation at eCO_2_. We also observed significant plasticity patterns in leaves under eCO_2_. Our findings highlight the importance of a thorough physiological understanding to inform innovative management practices aimed at mitigating the negative effects of climate change on plant N use efficiency.

## Introduction

Atmospheric carbon dioxide (CO_2_) concentrations are rising, reaching unprecedented levels (IPCC, 2023). This increase is primarily attributed to human activities such as industrial processes, fossil fuel combustion, and energy production. The rise in atmospheric CO_2_ concentrations exerts profound effects on plant development, resulting in alterations to growth dynamics, physiological processes, and broader ecosystem interactions (Aspray et al., 2023). Previous studies (Baslam et al., 2020; Ancín et al., 2024) have noted that elevated CO_2_ (eCO_2_) levels enhance photosynthetic efficiency, stimulate biomass accumulation, and affect nutrient distribution within plant tissues. As atmospheric CO_2_ continues to increase, it becomes imperative to identify and understand the key factors influencing plant growth under these conditions. Critical determinants, such as specific nutrient availability, particularly nitrogen (N), play a pivotal role in modulating plant responses to eCO_2_ and ultimately impact agricultural productivity (Porras et al., 2017). Additionally, persistent cloudy weather during crop growth stages—especially during the critical period—often leads to significant losses in grain quality and yield in rice due to low light conditions (Weng et al., 2017). As the Earth’s temperature continues to rise, altered weather patterns, such as increased cloud cover (Koshiro et al., 2022), will influence the amount of light available to plants (IPCC, 2023). Understanding how plant photosynthesis interacts with light patterns under a climate change scenario is essential to ensuring global food security (Murchie et al., 2009).

Nitrogen (N) remains a crucial nutrient for plants under both ambient and eCO_2_ conditions. The combined presence of the two main primary inorganic N sources—ammonium (NH_4_^+^) and nitrate (NO_3_^-^)—has a synergistic effect in promoting plant growth; however, the energy requirements for the assimilation of different N sources are markedly distinct (Bloom et al., 1992). Specifically, the photo-assimilation of NO_3_^–^ to NH_4_^+^ requires oxidation of NADPH or NADH and six reduced ferredoxins. Consequently, plants relying on NO_3_^–^ as an N source must efficiently allocate reductant generated during the light reactions of photosynthesis to meet the additional energy demands of NO_3_^–^ assimilation (Masclaux-Daubresse et al., 2010). Therefore, light intensity is likely to have a significant impact on plant preferences for N sources. Mitochondrial metabolism offers a promising avenue for enhancing N utilization efficiency (NUE) by modifying respiratory pathways, including the oxidative pentose phosphate pathway. These pathways can improve energy balance during N uptake (Foyer et al., 2011; Jauregui et al., 2017).

The influence of the N source extends to the plant’s carbon (C) balance. Plants can enhance their CO_2_ uptake rate during the *de novo* assimilation of NO_3_^-^ through photorespiration (Busch et al., 2018). This phenomenon is particularly critical for plants exposed to atmospheric eCO_2_, as several authors have indicated that NO_3_^-^ assimilation is significantly impaired under these conditions (Bloom et al., 2010, 2014; Jauregui et al., 2017). The assimilation of NO_3_^-^ requires NADP which is generated by photosynthesis However, under eCO_2_ conditions, C fixation is favored, and a greater proportion of reductant is utilized in this process. Additionally, as photorespiration decreases under eCO_2_, less malate is exported from the chloroplast to the cytosol, disrupting the NADH/NAD ratio in the cytosol, which is critical for NO_3_^-^ assimilation (Bloom et al., 2010). Other factors, such as reduced transpiration rates, further compound these metabolic imbalances (Jauregui et al., 2016). Remarkably, despite differences in N sources, C3 plants grown under higher than aCO_2_ show an overall depletion in their ionome, both in plant tissues and grains (Loladze, 2014). Specifically, Tcherkez et al. (2020) demonstrated that eCO_2_ reduces the concentrations of minerals in C3 plants (e.g., rice, wheat) by an average of 8%, while increasing the ratio of total non-structural carbohydrates to mineral content.

Rice, as a staple food for over half of the global population, plays a crucial role in food security, serving as a primary source of protein, carbohydrates, vitamins, and minerals. However, climate change poses significant challenges to rice production, with rising atmospheric CO_2_ concentrations emerging as a key factor impacting both crop yield and grain quality (Tcherkez et al., 2020). Studies indicate that higher CO_2_ levels increase carbohydrate content while reducing essential nutrients such as protein, zinc, and iron in rice grains, with profound implications for human health. Additionally, the reliance on chemical fertilizers in rice production exacerbates several environmental challenges. Excessive use of nitrogenous fertilizers can lead to nutrient imbalances, soil degradation, increased greenhouse gas emissions, and water contamination through runoff, resulting in eutrophication and harmful algal blooms. The interplay between climate change and rice cultivation techniques extends beyond the farm. A recent experiment (Xu et al., 2020) revealed that eCO_2_ stimulates a unique cluster of anaerobic microorganisms from the Burkholderiales family, which play a pivotal role in the release of N gas through NH_4_^+^ oxidation. This finding significantly enhances our understanding of the complex interactions between eCO_2_ levels and the N cycle in rice systems.

Our study investigates the interplay among light intensity (LL: 150 µmol m^−2^ s^−1^; and CL: 300 µmol m^−2^ s^−1^), N sources (nitrate; N-NO_3_ or ammonium nitrate; N-NH_4_NO_3_), and CO_2_ concentrations (410 and 700 ppm) on growth, photosynthetic efficiency, and N utilization in rice plants. Our research hypothesis posits that mixed nutrition improves plant responsiveness to eCO_2_ across varying light intensities compared to sole N-NO_3_ nutrition, due to its lower energy demands for N assimilation. The findings indicate that N-NO_3_ nutrition fails to enhance growth, photosynthetic efficiency, or N accumulation under aCO_2_ conditions, particularly in low light (LL) environments. Conversely, the application of N-NH_4_NO_3_ increases biomass accumulation and carboxylation efficiency under eCO_2_ conditions, supported by a corresponding increase in Calvin cycle-related sugar production. A decrease in leaf N and amino acid concentrations at eCO_2_, particularly in plants supplied with N-NO_3_, along with diminished ATP levels, suggests elevated energy demand for N assimilation. These findings highlight the critical role of N sources and light interactions in shaping plant adaptations to the challenges posed by climate change.

## Materials and methods

### Plant material and growth conditions

Seeds of *Oryza sativa* L. cv. Nipponbare were surface-sterilized with 2.5% (v/v) bleach and 0.02% (v/v) Triton X-100, then rinsed three times with sterile deionized water. Seeds were incubated at 30°C in the dark before transferring to 7.5-liter pots filled with nitrogen-free nursery culture soil (Kumiai Gousei Baido 4, JA, Tokyo, Japan). The plants were grown in a growth chamber (CFH-415; Tomy Seiko, Tokyo, Japan) under 12-hour light/dark cycles at 28/23°C, which contained a fluorescent lighting system as described by Inomata et al. (2018). Plants were watered twice a week.

The experimental design comprised four growth chambers with distinct environmental conditions to investigate plant responses to factorial conditions. A group of two chambers differed in light intensity (one at low light, LL, with 150 µmol m^-2^ s^-1^, and another at control light, CL, with 300 µmol m^-2^ s^-1^). Additionally, within each of the groups, one chamber functioned at atmospheric CO_2_ concentrations (410 ppm CO_2_; aCO_2_) while the other functioned at elevated CO_2_ concentrations (700 ppm CO2; eCO_2_). In each chamber, eighteen pots were divided into two nitrogen treatment groups: nine pots received N as Ca(NO_3_)_2_4H_2_O, while the other nine received NH_4_NO_3_ at 2.5 mM of N, with calcium being the element present at different concentrations between the nutrient solutions.

### Growth measurements

At the end of the experiment (50 days after germination; DAG), fresh leaf material from the fully expanded leaf was collected and stored in liquid nitrogen; then, this material was lyophilized using a freeze-drier (Testlar LyoQuest, Spain) and used for the metabolite and mineral measurements. Additionally, plant height was measured from the base of the tillers to the tip of the tallest leaf using a ruler. Leaf area was determined by scanning leaves and analyzing images using ImageJ software. Then, fresh weight was taken and dried in an oven at 60°C for 48 hours to determine the dry mass of each plant. The tiller number was counted manually. Roots were carefully washed with H_2_O to clean them. Fresh and dry weights were measured.

### Photosynthetic measurements

Gas exchange parameters were evaluated on fully developed apical leaves using a LI-COR 6400XT portable photosynthesis system (LI-COR Biosciences, Lincoln, NE, USA). Measurements were performed between 09:00 to 11:00 AM to reduce diurnal fluctuation. The chamber was set up with a 2 cm² standard leaf chamber and a red/blue LED illumination source. Environmental conditions within the chamber were regulated as follows: CO_2_ concentration at growth conditions (410 μmol mol−¹ or 700 μmol mol−¹) photosynthetic photon flux density (PPFD) at 1200 μmol m−² s−¹, and block temperature held at 25°C. Relative humidity was controlled to maintain leaf-to-air vapor pressure deficit between 1.0 and 1.5 kPa. Prior to measurements, the system was calibrated according to the manufacturer´s recommended protocols, including zeroing the CO_2_ and H_2_O analyzers and aligning the reference and sample gas analyzers. Leaves were acclimatized in the chamber for 5 minutes to stabilize gas exchange rates prior to data collection. Saturation CO_2_ assimilation rate (Asat), and transpiration rate (T_r_) were computed using the equations of von Caemmerer and Farquhar (1981). Afterwards, A/C^i^ response curves were produced by varying the chamber CO_2_ concentration from 50 to 1500 μmol mol−¹, with each step lasting 5 minutes to ensure equilibrium. Maximum carboxylation velocity (V_max_) and maximum electron transport rate (J_max_) were approximated by fitting the A/C_i_ data to the mechanistic model of C_3_; photosynthesis as outlined by Sharkey et al. (2007). All measurements were replicated on five individual plants per treatment, and data were adjusted for potential leaks and standardized to standard temperature and pressure conditions.

### ATP measurements

ATP quantification was conducted utilizing an ATP Determination Kit (A22066, Invitrogen) based on luciferase-based bioluminescence assay (Tatsumi et al., 1989). Approximately 50 mg of frozen rice leaf tissue was homogenized in 1 mL sonication solution (SONOP) buffer (0.372 g EDTA dissolved in 130 mL deionized distilled water H_2_O, adjusted to pH 10.9 with NaOH, then mixed with 370 mL of 96% ethanol) using a gentleMACSTM Dissociator. The mixture was centrifuged at 13,000×g, and protein concentration in the supernatant was determined using the Pierce™ BCA™ Protein Assay kit (Thermo Fisher Scientific). The protein concentration was adjusted to 300 µg/mL with SONOP buffer. Samples were further diluted 10-fold in 100 µM phosphate buffer, and ATP concentrations were determined using a calibration curve corrected for protein content and expressed as nmol per gram of protein.

### Mineral content

50 mg of lyophilized leaf sample was utilized, which is subjected to acid digestion utilizing a combination of concentrated HNO_3_ and H_2_O_2_ (5:1 volume ratio) within a temperature-regulated digestion block maintained at 180°C for 4 hours; subsequently, it was diluted with ultrapure water to achieve a specified volume. The digested samples were assessed for mineral composition using inductively coupled plasma/optical emission spectrometry (ICP/OES) on an iCAP 6500 Duo apparatus (Thermo Fisher Scientific, Waltham, USA), where the plasma is sustained at approximately 7000-8000K and sample introduction is facilitated through a nebulizer apparatus. Regarding the determination of C and N concentration (%), a separate aliquot of the same lyophilized sample undergoes dynamic flash combustion at 1800°C in an elemental analyzer (FlashEA1112, ThermoFinnigan) outfitted with a MAS200R autosampler, wherein the sample is entirely oxidized into elemental gases (CO_2_, H_2_O, and N_2_), which were subsequently separated via a chromatographic column and detected through thermal conductivity.

### Hormone profile

About 40 mg of lyophilized leaf sample was mixed with 80% methanol-1% acetic acid and internal standards, shaken at 4°C for an hour, then stored at -20°C overnight, and centrifuged before drying the supernatant. The dried residue was dissolved in 1% acetic acid, passed through an Oasis HLB column; for abscisic acid (ABA) quantification, the eluate was processed with 5% acetonitrile-1% acetic acid and separated using utra-high-performance liquid chromatography (UHPLC). To analyze cytokinins an Oasis MCX column was utilized, and after elution with 60% methanol-5% NH_4_OH, the basic fraction was dried, dissolved, and separated through UHPLC chromatography. Hormones were examined with a Q-Exactive mass spectrometer using targeted Selected Ion Monitoring, and their concentrations were determined using calibration curves generated with Xcalibur 4.0 and TraceFinder 4.1 SP1 software. Deuterium-labelled hormones were used as internal standards for quantification of each plant hormone.

### Metabolome profile

For the rice leaf metabolome analyses, all steps were adapted from the original protocol described by Fiehn et al. (2006). The ground dried samples (15 mg DW) were resuspended in 1 mL of frozen (-20°C) Water: Acetonitrile : Isopropanol (2:3:3) containing Ribitol at 4 pg/mL and extracted for 10 min at 4°C with shaking at 1500 rpm in an Eppendorf Thermomixer. Insoluble material was removed by centrifugation at 13,500 RPM for 10 min. Then, 50 µL were collected and 10 µL of myristic acid d27 at 30 pg/mL was added as an internal standard for retention time locking. Extracts were dried for 4 hours at 35°C in a SpeedVac and stored at -80°C.

Samples were taken out of -80°C, warmed 15 min before opening and SpeedVac dried again for 1.5 h at 35°C before adding 10 µL of 20 mg/mL methoxyamine in pyridine to the samples and the reaction was performed for 90 min at 30°C under continuous shaking in an Eppendorf thermomixer. 90 µL of N-methyl-N-trimethylsilyl-trifluoroacetamide (MSTFA, Thermo Scientific SAS) were then added and the reaction continued for 30 min at 37°C. After cooling, 100 µL were transferred to an Agilent vial for injection.

4 hours after derivatization 1 µl of sample was injected in splitless mode on an Agilent 7890B gas chromatograph coupled to an Agilent 5977A mass spectrometer. The column was an *Rxi-5SilMS* from Restek. An injection in split mode with a ratio of 1:30 was systematically performed for saturated compounds quantification. Oven temperature ramp was 60°C for 1 min, then increased at 10°C min−¹ to 325°C, held for 10 min. Helium constant flow was 1.1 mL min^-1^. Temperatures were the following: injector: 250°C, transfer line: 290°C, source: 230°C and quadrupole 150°C. The quadrupole mass spectrometer was switched on after a 5.90 min solvent delay time, scanning from 50-600 *m/z*. Absolute retention times were locked to the internal standard d27-myristic acid using the RTL system provided in Agilent’s MassHunter software. Retention time locking reduces run- to-run retention time variation. Samples were randomized. Fatty acid methyl esters mix (C8, C9, C10, C12, C14, C16, C18, C20, C22, C24, C26, C28, C30) was injected in the middle of the queue for external RI calibration.

Raw Agilent datafiles were analyzed with AMDIS http://chemdata.nist.gov/mass-spc/amdis/. The Agilent Fiehn GC/MS Metabolomics RTL Library (version June 2008) was employed for metabolite identification. Peak areas determined with the MassHunter Quantitative Analysis (Agilent) in splitless and split 30 modes. Resulting areas were compiled in one single Excel file for comparison. Peak areas were normalized to Ribitol and dry weight. Metabolite contents are expressed in arbitrary units (semi-quantitative determination). Additional information can be found in (Aranjuelo et al., 2015).

### Statistical analysis

Data were analyzed using one-way analysis of variance (ANOVA) followed by Tukey’s *post hoc* test to determine significant differences between groups. All statistical analyses were performed using R software.

Data from metabolomics were analyzed using MetaboAnalyst 6.0 software (Pang et al., 2024) through multivariate (MVA) and univariate (UVA) analyses. The MVA involved normalization (by sum), logarithmic transformation (by log_10_), and Pareto scaling, with principal component analysis (PCA) used for unsupervised modelling to ensure data quality and detect patterns. Discriminant models like PLS-DA and OPLS-DA were employed to identify group separations, while UVA utilized the t-test and Kruskal-Wallis test for group discrimination at a 95% confidence interval. The top 25 metabolites were identified using the Correlation and Partial Correlation Analysis based on Pearson’s R and considered significant if correlation coefficients were higher than ± 0.5. The fold change (FC) and percentage of variation (%) were calculated to determine differences between case and reference groups.

## Results

### Biomass accumulation

**Figure 1:**
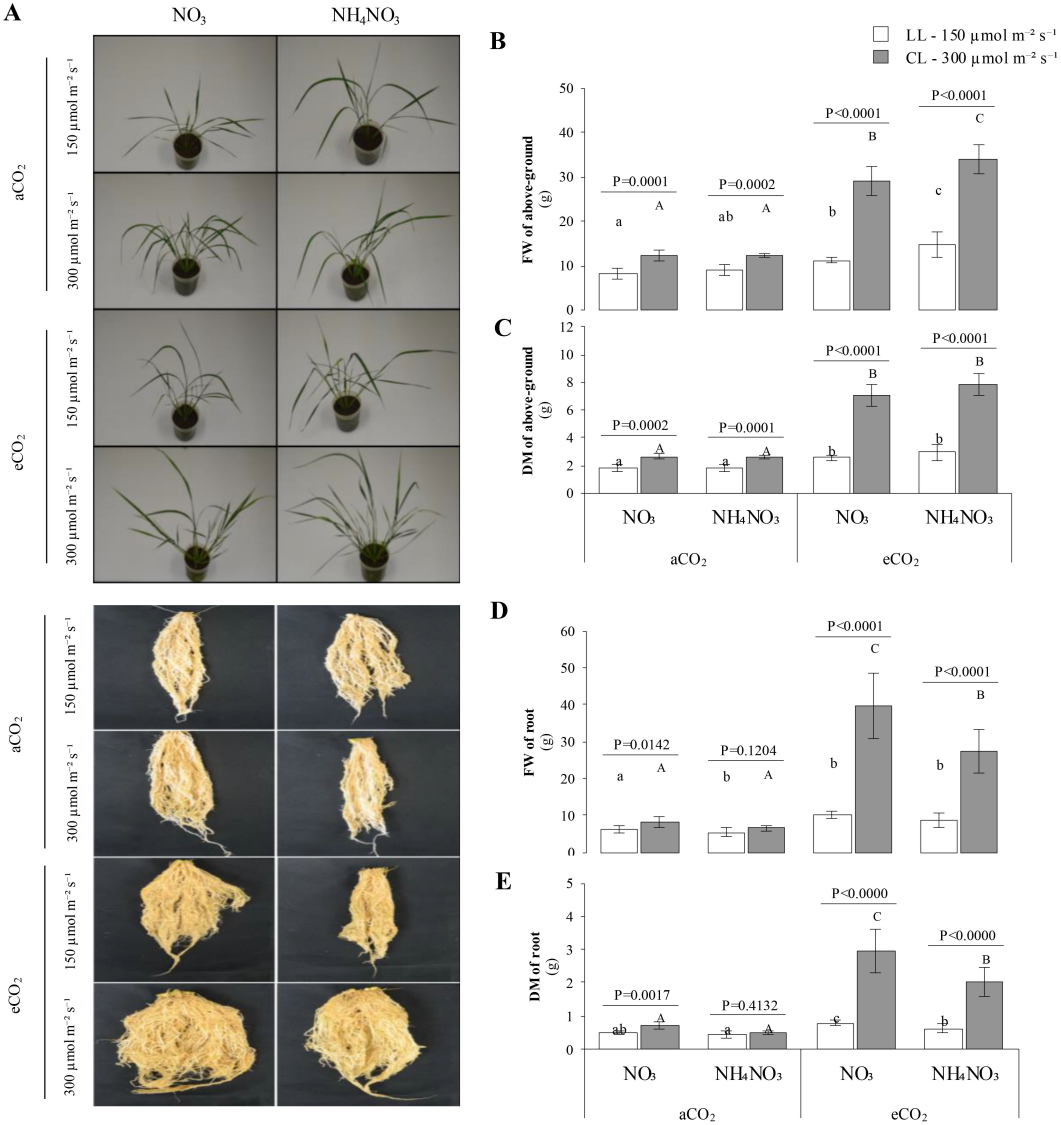
Represents the combined effect of nitrogen (N) form, irradiance, and CO2 concentration on rice plant growth. **(A)** shows representative images of rice plants grown under different combinations of ninitrate (NO3) or ammonium nitrate (NH4NO3), at two light intensities (150 µmol m^-2^ s^-1^ , white bars; or 300 μmol m^-2^ s^-1^, grey bars) and under ambient (aCO2) or elevated (eCO2) CO2 concentrations. **(B, C)** quantify the fresh weight (FW) and dry matter (DM), respectively, of the above-ground tissues (shoots) of these plants; similarly, **(D, E)** present the FW and DM of the root tissues. Bar graphs within **(B–E)** display means ± SD derived from 9 replicates. The lowercase letters indicate statistically significant differences within the LL (Low Light) treatment, while the uppercase letters denote significant differences within the CL (control Light) treatment.

The results indicate that the light intensity has a significant effect on aboveground biomass accumulation (**Figures 1A–C**). As expected, biomass accumulation increased significantly under higher irradiance at 300 µmol m^-2^ s^-1^ (CL), with *p* < 0.002. Notably, a substantial accumulation of aboveground biomass, by an order of magnitude, was observed when plants were exposed to eCO_2_ and low irradiance levels at 150 µmol m^-2^ s^-1^ (LL). At aCO_2_, no significant differences in fresh weight (FW) were observed between the two N sources at the same light intensity. However, under eCO_2_, FW showed a slight increase in plants supplemented with N-NH_4_NO_3_ compared to N-NO_3_ at both light intensities. For dry weight (DW), while aboveground biomass was higher under eCO_2_ than aCO_2_, no statistically significant differences between N sources were detected at the same light intensity. Regarding root biomass accumulation (**Figures 1A, D, E**), significant increases in both FW and DW were observed in plants grown with N-NO_3_ compared to N-NH_4_NO_3_ at both CO_2_ levels (*p-values* < 0.0142), regardless of light intensity. Additionally, root biomass accumulation increased significantly under eCO_2_ compared to aCO_2_. For N-NH_4_NO_3_ under aCO_2_, no differences were observed between light intensity treatments. However, higher root biomass was noted in LL × N-NO_3_ and in all treatments under eCO_2_ conditions.

**Figure 2:**
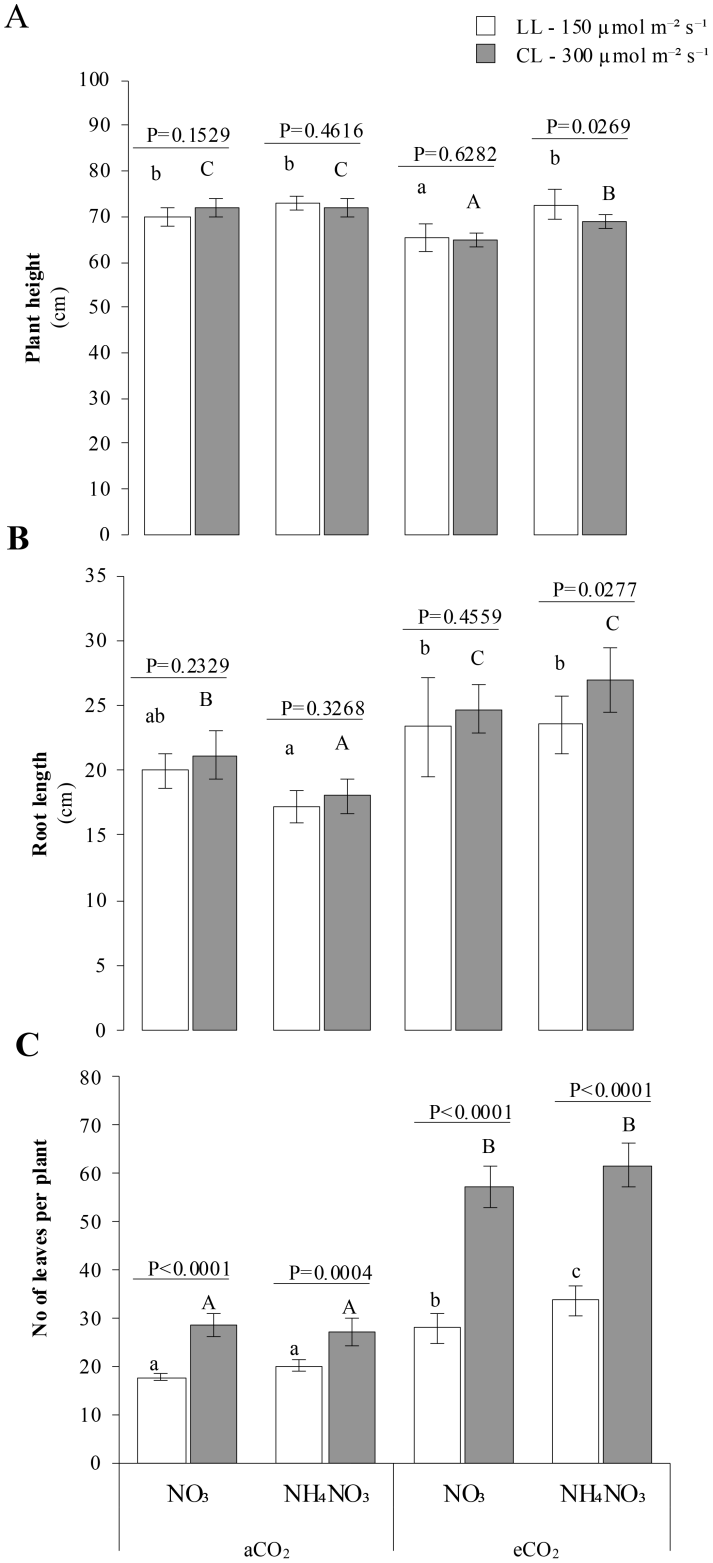
Represents the combined effect of N form, irradiance, and CO2 concentration on rice plant growth parameters. **(A)** A displays the plant height (cm) under various conditions, while **(B)** shows root length (cm). **(C)** represents the number of leaves per plant. In all panels, rice plants were grown with either nitrate (NO3) or ammonium nitrate (NH4NO3), at two light intensities (150 and 300 μmol m^-2^ s^-1^) represented by white and gray bars respectively, and under either ambient (aCO2) or elevated (eCO2) CO2 conditions. Bar graphs within **(A-C)** display means ± SD derived from 9 replicates. The lowercase letters indicate statistically significant differences within the LL (Low Light) treatment, while the uppercase letters denote significant differences within the CL (control Light) treatment.

Plants showed increased height (**Figures 2A**) under LL x eCO_2_ x N-NH_4_NO_3_ nutrition (*p-value* 0.0269). In contrast, no significant differences were observed between light treatments under aCO_2_ or in plants grown with N-NO_3_ under eCO_2_ (*p-values* > 0.05). The shortest plant heights were consistently observed in plants grown with N-NO_3_ and eCO_2_, irrespective of light conditions. Root length followed a similar trend: plants grown with N-NH_4_NO_3_ under eCO_2_ and CL displayed significantly greater root lengths (p-value 0.0269). Conversely, no significant differences in root length were observed between light treatments under aCO_2_ or in eCO_2_ × N-NO_3_ treatments. Plants grown under eCO_2_ × N-NH_4_NO_3_ exhibited shorter root systems at both light intensities (**Figure 2B**).

The number of leaves per plant (**Figure 2C**) was significantly higher in plants grown under CL compared to LL (*p-value <*0.0004). These differences were more pronounced under eCO_2_, but were also observed under aCO_2_. Furthermore, plants grown under eCO_2_ x N-NH_4_NO_3_ x LL had significantly higher leaf counts than those grown under LL x eCO_2_ x N-NO_3_, as indicated by the significance letters. The number of tillers per plant (**Figure 2D**) was significantly higher in plants grown under CL compared to LL (*p-value* < 0.0073). As with leaf count, these differences were more substantial under eCO_2_ and less pronounced under aCO_2_. Plants grown under eCO_2_ × N-NH_4_NO_3_ exhibited the highest number of tillers per plant across all light conditions.

### Plasticity of the leaves

**Figure 3:**
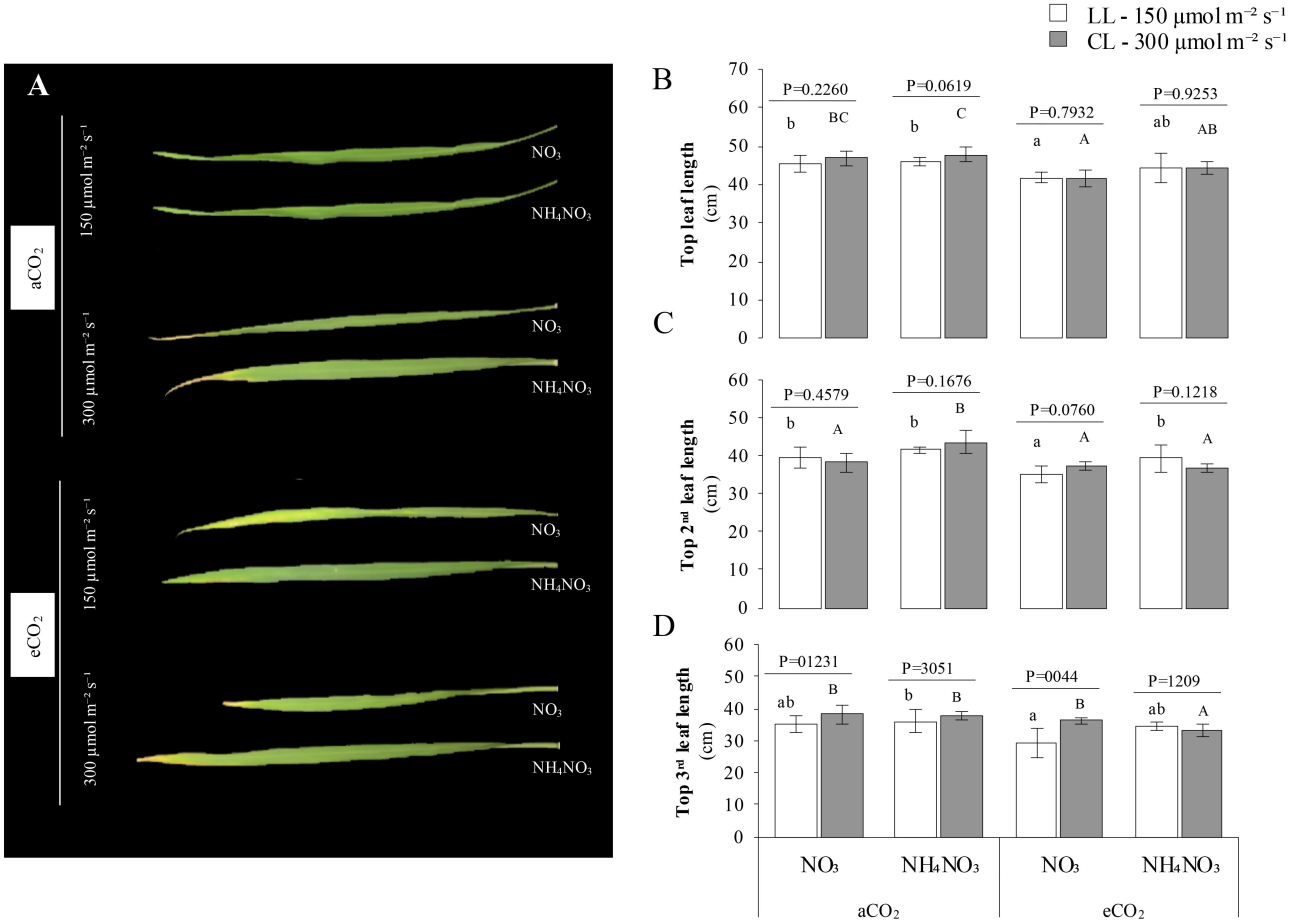
Represents the combined effect of N form, irradiance, and CO^2^ concentration on leaf length of rice plants. **(A)** shows representative images of the top, second, and third leaves of rice plants grown under different conditions. **(B-D)** present the length (cm) of the top, second, and third leaves respectively. In all panels, rice plants were grown with either nitrate (NO^3^) or ammonium nitrate (NH^4^ NO^3^), at two light intensities (150 and 300 μmol m^-2^ s^-1^) represented by white and gray bars respectively, and under either ambient (aCO^2^) or elevated (eCO^2^) CO^2^ conditions. Bar graphs within **(A-C)** display means ± SD derived from 9 replicates. The lowercase letters indicate statistically significant differences within the LL (Low Light) treatment, while the uppercase letters denote significant differences within the CL (control Light) treatment.

**Figure 3** illustrates the impact of the treatments on the width and length of the first, second, and third leaves of rice plants. The findings indicate that there was not a significant effect on the length of the flag leaf due to the treatments, in contrast to other growth parameters, as indicated by the *p-values*. Notably, the length of the flag leaf tended to increase under aCO_2_ in comparison to eCO_2_. Particularly, it was a notably higher flag leaf length at LL x aCO_2_ x N-NO_3_ compared to its counterpart under eCO_2_. Conversely, the width was influenced by the N treatment: plants supplied with N-NO_3_ exhibited a significantly narrower flag leaf width than those with N-NH_4_NO_3_ (*p-value* < 0.0024). Moreover, under conditions of LL x eCO_2_, plants exhibited a notably wider width when subjected to a combination of N-NH_4_NO_3_ compared to solely N-NO_3_. Similarly, the impact of the treatments on the 2^nd^ leaf length was found to be non-significant when comparing its counterpart at different LL, as denoted by the *p-values*. A larger 2^nd^ leaf was observed under HI x aCO_2_ conditions when N-NH_4_NO_3_ was provided compared to N-NO_3_ nutrition. Conversely, under LL x eCO_2_, N-NH_4_NO_3_ nutrition led to an increase in 2^nd^ leaf length in comparison to N-NO_3_. The extension patterns of 2^nd^ leaf length were comparable across different light intensities, showing no discernible differences. A notable increase in width was noted in plants receiving N-NH_4_NO_3_ as opposed to N-NO_3_, specifically under eCO_2_ and LL conditions. Conversely, the 3^rd^ leaf length and width exhibited significant variations due to the treatments. Plants grown under HI conditions displayed longer and wider leaves compared to those under LL conditions, particularly evident under eCO_2_ with N-NO_3_ at both light intensities (*p-value* < 0.044 for LL and < 0.0001 for HI). Furthermore, a reduction in the length and width of the upper third leaf was observed under eCO_2_ and CL conditions when N-NH_4_NO_3_ was supplied.

### Photosynthetic parameters and ATP

**Figure 4:**
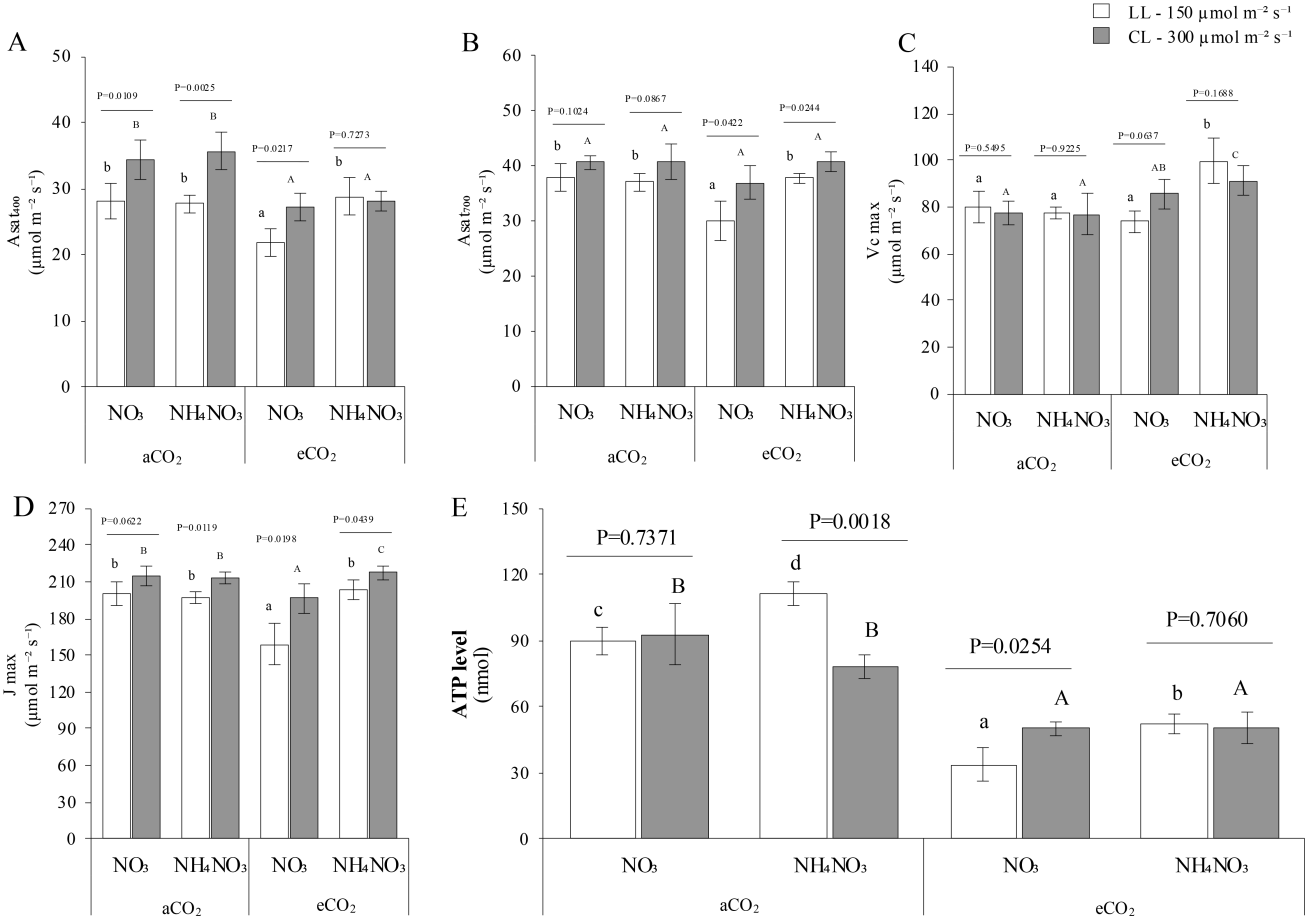
Represents the combined effect of N form, irradiance, and CO_2_ concentration on leaf length of rice plants. **(A, B)** shows assimilation rate (Asat) at growing CO_2_. **(C, D)** the maximum carboxylation velocity (Vmax) and themaximum electron transport rate (Jmax) repectively. **(E)** represents adenosine triphosphate (ATP) levels. In all panels, rice plants were grown with either nitrate (NO_3_) or ammonium nitrate (NH_4_NO_3_), at two light intensities (150 and 300 μmol m^-2^ s^-1^) represented by white and gray bars respectively, and under either ambient (aCO_2_) or elevated (eCO_2_) CO_2_ conditions. Bar graphs within **(A-D)** display means ± SD derived from 5replicates while from ATP was derived from 3-4 replicates. The lowercase letters indicate statistically significant differences within the LL (Low Light) treatment, while the uppercase letters denote significant differences within the CL (control Light) treatment.

As anticipated, net photosynthesis at growing CO_2_ conditions (410 ppm for aCO_2_ and 700 ppm for eCO_2_; **Figures 4A, B**) was markedly higher at HI. Notably, higher net photosynthetic rates at eCO_2_ were observed only for N-NH_4_NO_3_, irrespective of light levels. Besides, the phenomenon of photosynthetic acclimation to eCO_2_—reduction of the theoretically potential fixation capacity–was specifically evident in N-NO_3_ plants. Hence, plants grown with eCO_2_ x N-NH_4_NO_3_ exhibited the highest velocity of carboxylation (Vc_max_; **Figure 4C**) at eCO_2_ x N-NH_4_NO_3_ at both light levels. Interestingly, no significant differences were detected between light treatments, indicating potential constraints on carbon fixation capacity, such as the photosynthetic electron transport capacity (J_max_; **Figure 4D**). It is noteworthy that plants supplied with N-NO_3_ exhibited the lowest J_max_ under eCO_2_ at both light levels. Furthermore, the J_max_ at CL showed near-significance for N-NO_3_ x aCO_2_ compared to plants at different light levels, and increased at CL for aCO_2_ x N-NH_4_NO_3_, eCO_2_ x N-NO_3_, and eCO_2_ x N-NH_4_NO_3_ (*p values* of 0.0119, 0.0198, and 0.0439, respectively). The transpiration rates (**Supplementary Figure S1B**) exhibited a comparable pattern to g_s_, but the intriguing pattern of contrasting results in LL conditions was not statistically significant on this occasion. Finally, ratio of intercellular CO_2_ to aCO_2_ (the Ci/Ca ratio; **Supplementary Figure S1C**) increases in CL comparing to LL in aCO_2_ x N-NO_3_, aCO_2_ x N-NH_4_NO_3_ and eCO_2_ x N-NO_3_, as denoted by the *p-values*. Moreover, the ratio of eCO_2_ x N-NO_3_ was found to be the lowest at LL, whereas aCO_2_ x N-NO_3_ exhibited the highest ratio at CL. The results indicate that the amount of light exposure profoundly impacts the ATP levels in leaves (**Figure 4E**). Interestingly, higher ATP levels were seen in plants with aCO_2_ levels compared to eCO_2_ levels in all experimental conditions. Additionally, under N-NH_4_NO_3_ x aCO_2_, the ATP content is higher at CL than at LL (*p-value* 0.018); at eCO_2_, ATP levels decreased at LL in N-NO_3_ fed plants (*p-value* 0.0254).

### Ionomics

**Figure 5:**
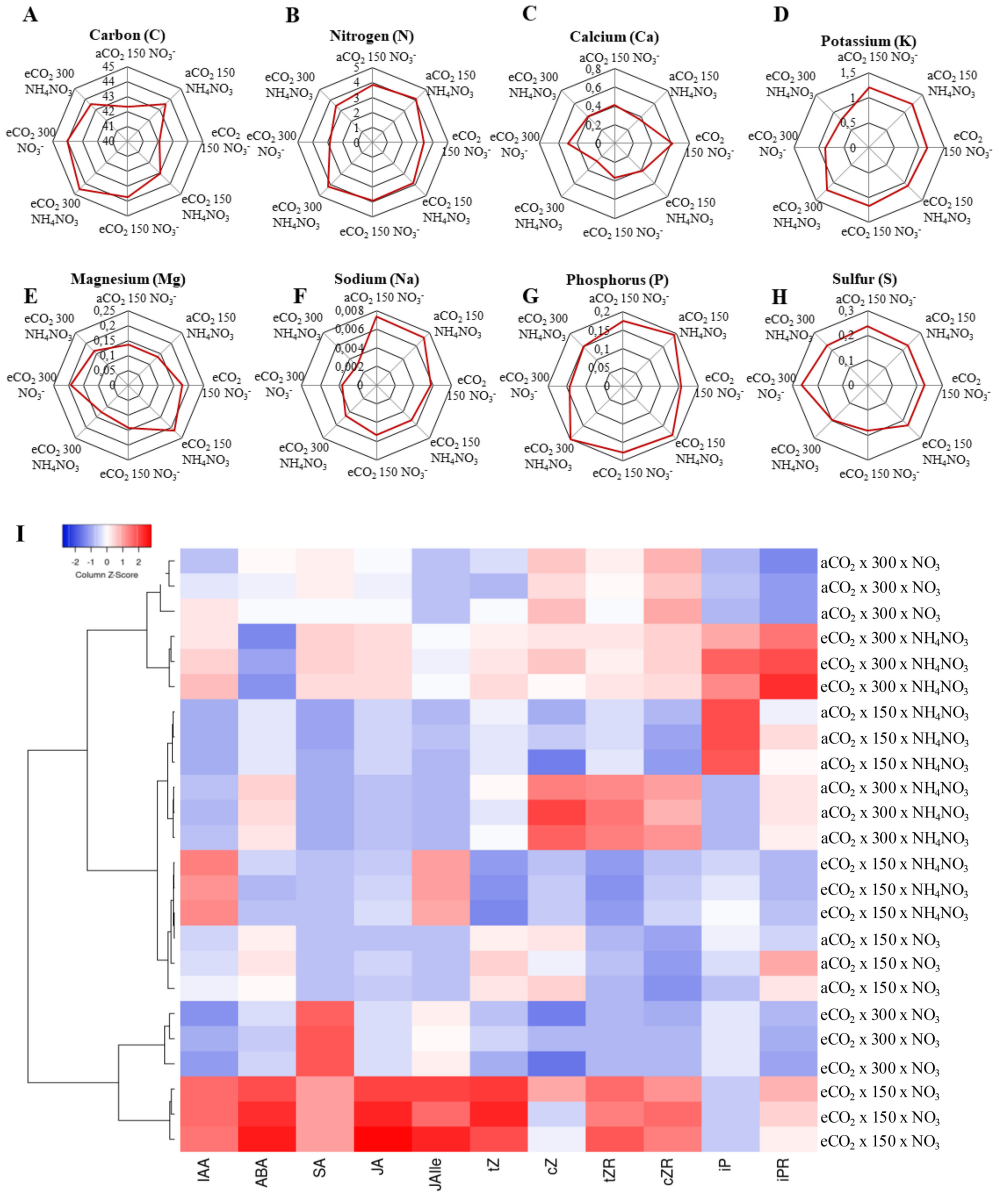
Illustrates the impact of various N forms (NO_3_ or NH_4_NO_3_) on selected leaf minerals **(A–H)** and phytohormones **(I)** contents of rice plants grown under contrasting irradiances (150 µmol m^−2^ s^−1^ or 300 mmol m^−2^ s^−1^) and cultivated in either ambient (aCO_2_) or elevated (eCO_2_) CO_2_ conditions. The values presented are means ± SD derived from 3 replicates.

**Figure 5** illustrates the impact of the treatments on selected leaf mineral content; the entire ionome profile is presented in **Supplementary Figure S1**. The findings indicated a significant rise in the total leaf carbon (C) content of plants grown under CL conditions at eCO_2_ levels (**Figure 5A**). Additionally, N-NH_4_NO_3_ showed a trend towards enhancing the C content. The N content experienced a significant reduction in plants grown under N-NO_3_ and eCO_2_ levels regardless of light intensity. Additionally, a decrease in N content was noted in CL conditions when exposed to eCO_2_ x N-NH_4_NO_3_ compared to its counterpart at LL. The potassium (K) content exhibited a remarkable decrease in plants cultivated under CL x eCO_2_, in clear contrast to LL x eCO_2_, at both N regimes. No discernible distinctions were observed for alternative combinations. An evident rise in calcium (Ca) concentrations was noted in plants grown under LL x eCO_2_ x N-NO_3_ compared to its counterpart at CL. Furthermore, a decline in Ca levels was identified in CL x aCO_2_ x N-NH_4_NO_3_ compared to the same combination at LL. Elevated levels of magnesium (Mg) were observed in plants subjected to increased LL x eCO_2_ x N-NH_4_NO_3_ compared to CL conditions. Moreover, sodium (Na) concentrations were notably higher in plants grown under LL conditions compared to CL in all cases. The phosphorus (P) content tended to increase with N-NH_4_NO_3_, but it remained comparable at CL x eCO_2_. Additionally, at eCO_2_ x N-NH_4_NO_3_, plants had higher P with LL. Finally, the sulfur (S) content decreased at CL compared to LL in aCO_2_ for both N treatments.

### Hormone profile

A hormone profile was conducted in leaves in order to capture possible profile that explains the physiological responses recorded; the results are represented in a heatmap (**Figure 5B**). First, the result that stands out is the notable rise across multiple hormonal markers (excluding cis-Zeatin (cZ), isopentenyladenine (iP), isopentenyladenosine (iPR)) in the experimental conditions involving the LL x eCO_2_ x N-NO_3_. The only other treatment that demonstrated an overall rise in the hormone levels, albeit less pronounced— with increases in almost all hormones except for ABA— was CL x eCO_2_ x N-NH_4_NO_3_. In relation to the other two treatments in eCO_2_, there is a significant rise in salicylic acid (SA) observed for CL x eCO_2_ x N-NO_3_ and, a slight rise in Indole-3-acetic acid (IAA) and jasmonoyl-isoleucine (JAlle) for LL x eCO_2_ x N-NH_4_NO_3_. In terms of the hormone levels in plants under aCO_2_ conditions, what is notable is the overall decrease in hormones with the exception of certain specific groups: for CL x aCO_2_ x N-NH_4_NO_3_ the zeatin cZ, the zeatin riboside (tZR) and cis-zeatin riboside (cZR) and cZR shows a significant increase, while a slight increase is observed for CL x aCO_2_ x N-NO_3_. iP has been largely accumulated in LL x aCO_2_ x N-NH_4_NO_3_.

### Metabolomics

Metabolites that were verified by the standard and tentatively identified through spectral similarity scores were the sole entities considered for inclusion in the statistical examination, which encompassed 108 metabolites.

### Single factor analysis

**Figure 6:**
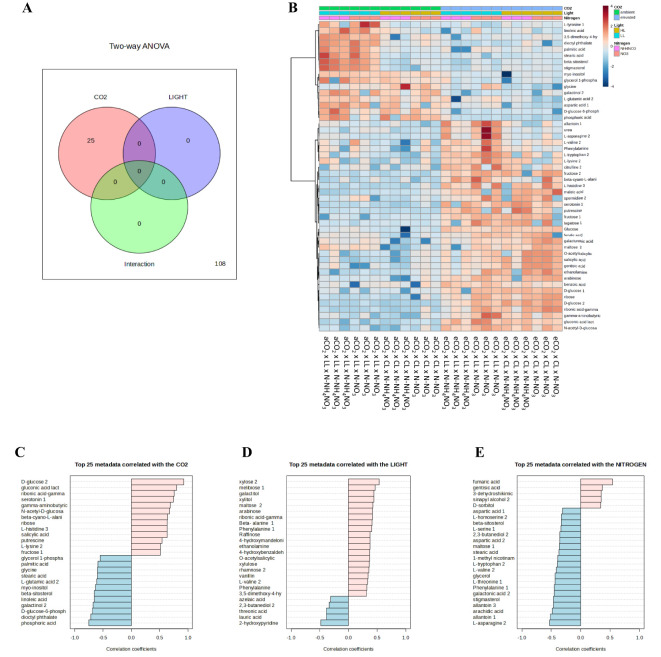
Illustrates the impact of various nitrogen forms (nitrate or ammonium nitrate) on selected leaf metabolites varying between treatments using **(A)** Venn diagram, **(B)** heatmap, and **(C–E)** the top 25 varying metabolites of rice plants grown under irradiances of 150 µmol m^-2^ s^-1^ (depicted by white bars) and 300 µmol m^-2^ s^-1^ (represented by gray bars), and cultivated in either ambient (aCO_2_) or elevated (eCO_2_) CO_2_ conditions. The values presented are means ± SD derived from 3 replicates.

When examining the metabolites influenced by the different treatments by analyzing the metabolites using 2-way ANOVA in Venn diagrams (**Figure 6A**), it was observed that only the CO_2_ treatment led to significant differences, indicating the greatest impact of this treatment. **Figure 6B** summarizes how the top 25 metabolites respond to increased levels of CO_2_, light, and different N sources; the entire heatmap with the entire metabolome can be seen in **Supplementary Figure S2**.

The foremost metabolites whose content was altered by the treatments CO_2_, light, and N are depicted in **Figures 6C–E**; only metabolites that changed by a correlation coefficient greater than 0.5 were deemed significant. The compounds that escalated with the CO_2_ treatment were: D-glucose 2, gluconic acid, ribonic acid-gamma, serotonin, gamma-aminobutyric, N-acetyl-D-glucose, beta-cyno–alanine, ribose, L-histidine, salicylic acid, putrescine, L-lysine, and fructose. Furthermore, the leading metabolites that were diminished with the CO_2_ treatment were: phosphoric acid, dioctyl phthalate, D-glucose-6-phosphate, galactinol, linoleic acid, beta-sitosterol, myo-inositol, L-glutamic acid, stearic acid, glycine, palmitic acid, and glycerol 1-phosphate. Concerning illumination and nitrogen, only marginally significant findings were found: In the case of light, only xylose 2 correlated positively, and in the case of nitrogen, only fumaric acid correlated positively, while L-asparagine and allantoin correlated negatively.

Through the categorization of the metabolites using analyses of variance of 1-way and false discovery rate, 16 metabolites were identified as significant (**Supplementary Figure S3**). The pattern of accumulation of metabolites changes differentially across the treatments. D-glucose exhibits a substantial increase under eCO_2_ levels in comparison to aCO_2_. Additionally, under LL x eCO_2_ x N-NO_3_, the quantity of D-glucose is lower than in the remaining treatments. In line with D-glucose, gluconic acid lactone (aerobic oxidation of glucose) shows a notably higher content at eCO_2_ relative to aCO_2_. The concentration of ribonic acid-gamma-lactone increased at eCO_2_ compared to aCO_2_. Moreover, under LL x eCO_2_ x N-NO_3_ conditions, the concentration is lower compared to the other treatments at eCO_2_. Serotonin displays a similar trend to ribonic acid-gamma-lactone; however, the variability under LL x eCO_2_ x N-NO_3_ hinders the distinction from aCO_2_. Furthermore, at LL x aCO_2_, the serotonin is accumulated at N-NH_4_NO_3_ nutrition compared to its counterpart. Both benzoic acid and salicylic acid tend to accumulate higher amounts at eCO_2_ compared to aCO_2_ counterpart. Additionally, under both CO_2_ levels and CL, rice plants at N-NO_3_ accumulated higher levels than at N-NH_4_NO_3_. In contrast, linoleic acid and phosphoric acid tend to gather lower quantities at eCO_2_ compared to aCO_2_. The level of the amino acid glycine rises under CL compared to LL at aCO_2_; however, at eCO_2_, while the glycine content is higher with N-NO_3_ nutrition under LL, it is significantly reduced under CL. The metabolites beta-sitosterol, stigmasterol, stearic acid, and palmitic acid collectively display a similar trend: They show significantly higher contents at LL x aCO_2_ compared to the other conditions. Slightly lower accumulation was found in CL x aCO_2_ x N-NH_4_NO_3_ compared to its counterpart with N-NO_3_ for beta-sitosterol. No other major distinctions were observed under eCO_2_. In contrast, fumaric acid exhibits the lowest levels at LL x aCO_2_, at both nitrogen regimes, while its level is stable across the other treatments. The levels of L-asparagine and maltose did not follow any of the patterns described above. Plants tend to accumulate higher amounts of L-asparagine at eCO_2_ under low light, reaching the peak with N-NO_3_. Additionally, at aCO_2_ under LL x N-NH_4_NO_3_, the levels are higher than in the other aCO_2_ treatments. The pattern of maltose is partially obscured by variability in two treatments, but, CL x aCO_2_ x N-NH_4_NO_3_ tends to displays the lowest levels. The highest maltose levels are observed at eCO_2_ under LL with N-NO_3_.

### Exploratory group analysis

A Partial Least Squares Discriminant Analysis (PLS-DA) is a supervised dimensionality reduction method that, unlike PCA, incorporates class labels in the analysis. First, the SPLS plot allows grouping the results in 2 dimensions, with component 1 explaining 16.1% of the variability and component 2 accounting for 9% (**Supplementary Figure S4**). Moreover, these clusters are distinctly separated from the eCO_2_ treatments.

To understand the core metabolic changes that lead to the observed traits, it is necessary to study how different factors interact in detail. This is why the Simultaneous Component Analysis (ASCA) method, which merges analysis of variance (ANOVA) with Simultaneous Component Analysis (SCA), was chosen. By using this method, relationships between treatments can be explored with a significance level of *p-value* < 0.05. An ASC analysis was conducted to create leverage and squared prediction error (SPE) plots to pinpoint metabolites that conform to or deviate from ASCA patterns. Metabolites with a leverage threshold of 0.9 and an alpha threshold of 0.05 high leverage and low SPE values are considered well-modeled and are thus identified as influential compounds. Additionally, PLS aids in reducing dimensionality and can be tailored for feature selection and classification purposes. When contrasting the treatments CO_2_ and light (**Supplementary Figure S5A**), the organic compounds N-acetyl-D-glucosamine, gluconic acid lactone, GABA and D-glucose were effectively simulated by factor CO_2_; xylose, arabinose, beta-alanine and ethanolamine were effectively simulated by factor light; finally, L-asparagine and salicylic acid were effectively simulated by their interaction. By contrasting CO_2_ and N (**Supplementary Figure S5B**), the substances N-acetyl-D-glucosamine, gluconic acid lactone, GABA and D-glucose were effectively simulated by the treatment CO_2_; the substances fumaric acid, allantoin 1, gluconic acid 2, arachidic acid and allantoin 2 were effectively simulated with N; finally, the substances galacturonic acid, glycine and beta-sitosterol acid were effectively simulated with the interaction. By contrasting illumination and nitrogen using an ASC analysis, distinctions can be identified (**Supplementary Figure S5C**). The substances xylose 2, arabinose, beta-alanine 1 and ethanolamine were effectively simulated by the factor light; the substances fumaric acid, allatoin 1, gluconic acid 2, arachidic acid and allantoin 3 were effectively simulated by the factor nitrogen; the substances threonic acid 1,4-lactone, 2,3-butanediol, proline and 2,3- butanediol 2 were effectively simulated by the interaction.

## Discussion

Despite its importance, limited information exists regarding how light intensity interacts with other factors influencing crop performance, such as fertilization and CO_2_ levels (Aspray et al., 2023). This gap is significant because crop development is heavily impacted by reduced light intensity, which plays a crucial role in yield formation. In Southeast Asia, for instance, early and late monsoon periods are often characterized by prolonged overcast conditions. Building on the findings of Weng et al. (2017), and assuming that 47.5% of solar radiation falls within the PAR range, we simulated the lower irradiance conditions observed in this study at 8 MJ per day, corresponding to 150 µmol m^-2^ s^-1^ PAR. Notably, many days with solar irradiance below 8 MJ per day have been reported by Weng et al. (2017). According to Venkateswarlu et al. (1977), insufficient light negatively affects all stages of rice growth, reducing tillers and panicles during vegetative growth, while disrupting biochemical and physiological processes that lead to fewer spikelets, lower grain weight, and diminished grain quality during the reproductive stage. Our research builds on these findings, exploring not only the impact of low light but also the interplay between light intensity, CO_2_ levels, and N supplementation. Specifically, our study was designed to investigate how different forms of N supplementation influence rice plant growth across varying CO_2_ levels and light intensities, including LL conditions. The results revealed that N source significantly influences plant growth and physiological responses under changing climate conditions, particularly in LL environments. Notably, eCO_2_ positively affected above-ground biomass under LL conditions, with the effect being more pronounced when N-NH_4_NO_3_ was used as the fertilizer. Additionally, both leaf count and tiller number increased under eCO_2_ across light regimes, with greater enhancement observed in plants supplemented with N-NH_4_NO_3_. These findings underscore the importance of considering not only the direct effects of climate change but also the pivotal role of N fertilization in mediating plant responses to varying light intensities.

Previous studies have shown that while plants initially experience a boost in photosynthesis due to increased substrate availability under eCO_2_, this enhancement tends to diminish over time (Ancín et al., 2024; Tcherkez et al., 2024). This phenomenon, known as photosynthetic acclimation, was first described by Webber et al. (1994). The accumulation of carbohydrates, particularly glucose, fructose, and starch, disrupts the balance of N compounds, including amino acids, and leads to a reduction in protein levels such as Rubisco. This reduction, in turn, lowers photosynthetic capacity in plants exposed to eCO_2_ levels (Tcherkez et al., 2020). The current study demonstrated that crop responsiveness to N fertilization strategies is CO_2_ dependent, consistent with previous findings in other crops (Porras et al., 2017; Domiciano et al., 2020; Collado-González et al., 2022). Under aCO_2_, no significant variations were observed among the different N fertilization regimes, regardless of light intensity. However, under eCO_2_, plants fertilized with N-NH_4_NO_3_ exhibited a significant enhancement in photosynthetic efficiency, irrespective of light intensity. In contrast, plants supplemented with N-NO_3_ experienced a reduction in their carbon fixation ability due to adaptation to higher CO_2_ levels, which led to decreased carboxylation velocity and, particularly, electron transport capacity. This was accompanied by a reduction in ATP levels under LL x eCO_2_ x N-NO_3_, indicating severe energy constraints in these plants. As noted by several researchers (Bloom, 2010; Jauregui et al., 2015, 2017; Rubio-Asensio and Bloom, 2017), the decrease in NO_3_^-^ assimilation at higher CO_2_ levels is attributed to decreased photorespiration, which disrupts the C/N balance and ultimately hampers carbon assimilation. However, Andrews et al. (2019) have argued that eCO_2_ does not impede NO_3_^−^assimilation in C_3_ plants. It is worth noting that the study by Andrews et al. (2019) was conducted under high light conditions, exceeding ∼950 μmol photons m^−2^ s^−1^ of PAR, whereas Bloom´s and Jauregui’s research (Bloom, 2010; Jauregui et al., 2015, 2017; Rubio-Asensio and Bloom, 2017) was based on lower light levels of ∼350 µmol m^–2^ s^–1^ of PAR. Our findings suggest that low light levels, such as those used in this study, exacerbate energy constraints under eCO_2_, particularly in plants supplied with N-NO_3_ fertilization.

Various photosynthetic proteins, particularly those in the outer membrane of the chloroplast, are influenced by Ca^2+^ and play critical roles in responding to environmental cues and regulating photosynthesis. For instance, Hou et al. (2019) demonstrated that Ca^2+^ application is a target factor in photosynthetic responses to low light levels, highlighting its role in regulating gas exchange through fluid movement. In our experiment, an increase in Ca^2+^ levels was observed under LL conditions, but not in plants receiving N-NH_4_NO_3_. This is notable given the significant decrease in Ca²^+^ levels in leaves reported in a meta-analysis by Loladze (2014). The observed accumulation of Ca²^+^ under eCO_2_ × N-NO_3_ further supports the notion that reduced reductant power severely restricts photosynthesis and overall plant performance under these conditions. Recognizing the potential signaling role of minerals such as Ca^2+^, we expanded our study to analyze the phytohormone profile in leaves to explore the intricate interplay between mineral signaling and hormonal regulation in plant physiology. As anticipated, a pronounced increase in the phytohormone profile was observed under LL × eCO_2_ × N-NO_3_, indicating a stress adaptation response. This pattern was not evident under LL × aCO_2_ × N-NO_3_ or LL × aCO_2_ × N-NH_4_NO_3_, further underscoring the unique interplay between light intensity, CO_2_ levels, and N fertilization. Considering the significant changes in the hormone profile and the N limitations, it is not surprising that root biomass growth and plant height were greatest under eCO_2_ x N-NO_3_ at both light regimes. This finding suggests a strategic growth adjustment aimed at compensating for N deficiency in these plants. Our research underscores the complexity of plant adaptation mechanisms under future [CO_2_] levels and highlights the necessity of considering multiple interacting variables when modeling plant responses to environmental stress.

The significant impact of CO_2_ on the metabolome in the current study is evident from the enrichment analysis (**Supplementary Figure 2**), particularly in pathways such as glucose-fructose-mannose, arginine, and proline metabolism, which reflect a higher C status in rice plants. The metabolome exhibited minimal changes due to light treatments or N fertilization alone, indicating potential interactions among factors. To pinpoint the specific pathways affected by the combination of LL x N-NO_3_, a one-factor ANOVA was performed. First, we found that although sugars such as glucose and glucono-delta-lactone increased under eCO_2_ levels, their concentrations decreased at LL x N-NO_3_. Serotonin, known to function as both a potent antioxidant and a signaling molecule regulating root system structure (Pelagio-Flores et al., 2011), was detected in high levels under LL x eCO_2_ x N-NO_3_. This corresponds to the expansion of the root system observed in our study and likely reflects either a stress-induced response or an adaptation mechanism in the plants. Additionally, benzoic acid and salicylic acid concentrations were highest under LL x eCO_2_ x N-NO_3_, aligning with the profound hormonal modifications reported under similar conditions by others (Li et al., 2022). The increase in glycine concentration under LL x eCO_2_ x N-NO_3_ likely reflects a decrease in photorespiration rates caused by eCO_2_, compounded by limited reductant power available under LL conditions (Busch et al., 2018). Similarly, L-asparagine, which is indirectly related to photorespiration, followed a similar pattern to glycine under LL x eCO_2_ x N-NO_3_. These results mirror changes observed under aCO_2_ at LL compared to aCO_2_ at CL conditions (Rosa-Téllez et al., 2024). Overall, the physiological and hormonal changes recorded in our study illustrate the pronounced photosynthetic acclimation to eCO_2_ observed in N-NO_3_-fertilized plants.

Our findings emphasize the critical relationship between light intensity and various metabolic processes, particularly N metabolism, under eCO_2_ conditions. When plants are supplied only with N-NO_3_ as a nutrient, their ability to maintain optimal N levels becomes restricted, supporting the notion that hindered NO_3_^-^ assimilation arises from a decrease in reductant power. In the restrictive LL treatment, plants underwent extensive hormonal signaling modifications to manage stress and enhance adaptability. These changes included alterations in tillering, leaf development, and shoot-to-root ratios, which collectively represent a sophisticated interplay among metabolic pathways aimed at overcoming these challenges.

## Data Availability

The original contributions presented in the study are included in the article/**Supplementary Material**. Further inquiries can be directed to the corresponding authors.
