# Supplementary material for: Nitrogen fertilization form and energetic status as target points conditioning rice responsiveness to elevated [CO2]
Source: Front Plant Sci. 2025 Mar 11;16:1517360. doi: 10.3389/fpls.2025.1517360 (PMC11933000; doi:10.3389/fpls.2025.1517360)
Supplement: Supplementary file 1 [file DataSheet1.pdf]

Supplemental Figures

Supplemental Figure S1

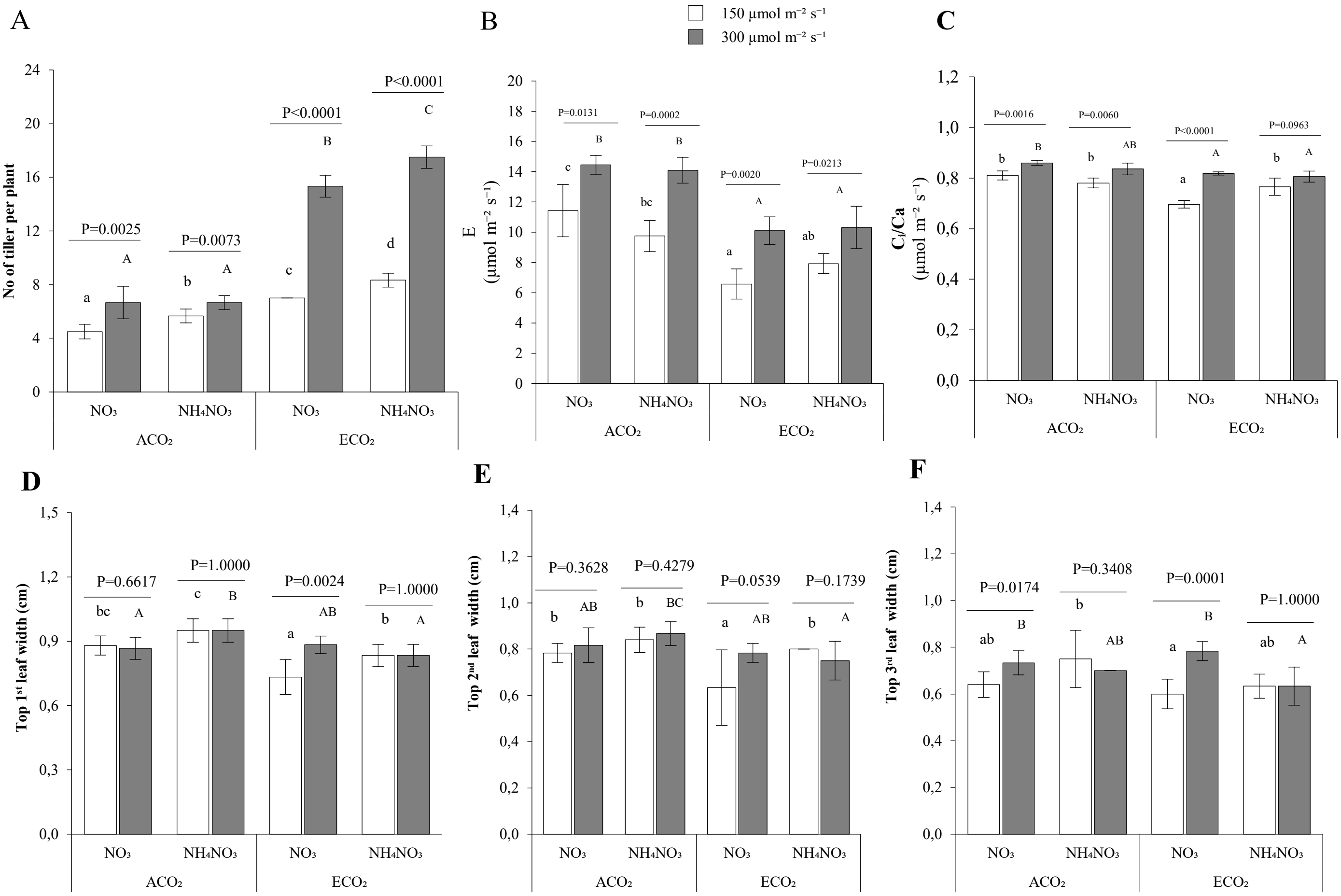

Fig. S1 illustrates the impact of various N forms (NO_3 or NH_4NO_3) on the numbers of tillers per plant, transpiration (E), ratio Ci/Ca, and top, second and third leaf width of rice plants grown under irradiances of 150 µmol m^{-2} s^{-1} (depicted by white bars) and 300 µmol m^{-2} s^{-1} (represented by gray bars), and cultivated in either ambient (ACO_2) or elevated (ECO_2) CO_2 conditions. The values presented are means ± SD derived from 9 replicates.

Supplemental Figure S2

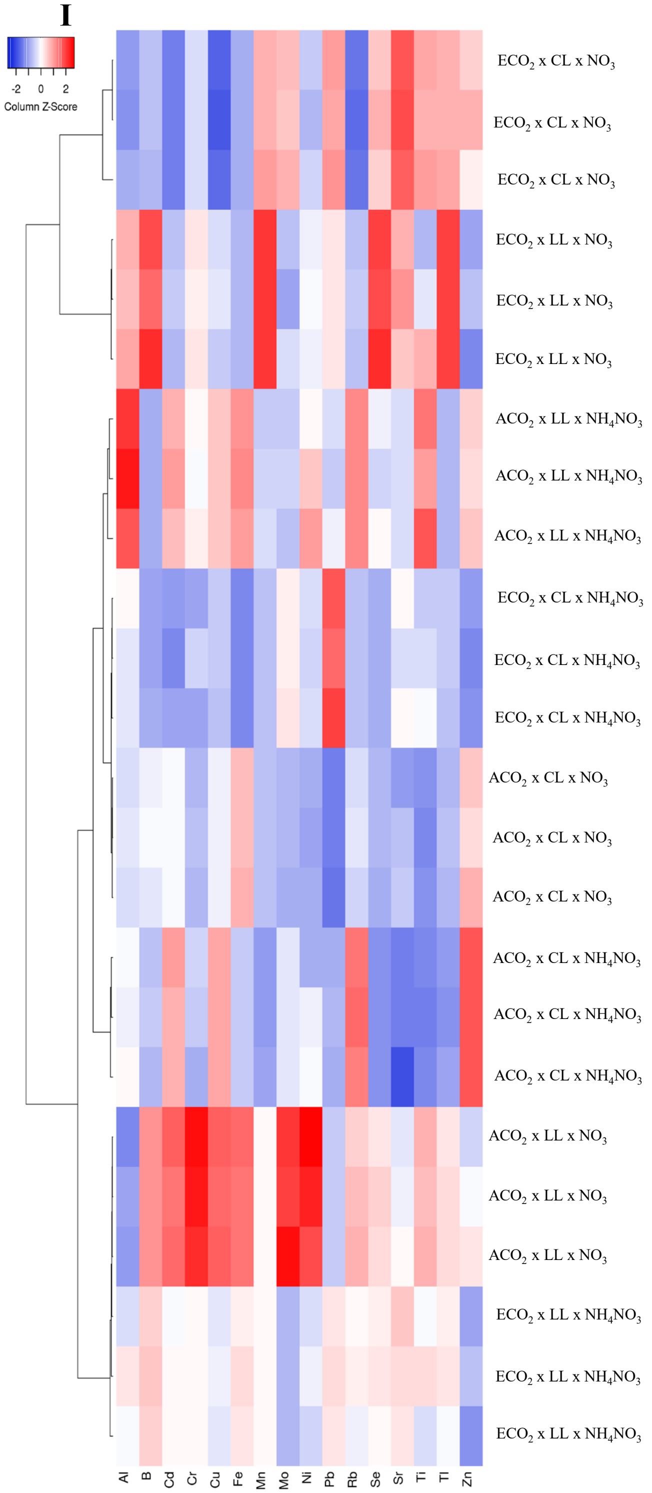

Al, B, Cd, Cr, Cu, Fe, Mn, Mo, Ni, Pb, Rb, Se, Sr, Ti Tl, Zn

Fig. S5 illustrates the impact of various N forms (NO_3 or NH_4NO_3) on selected leaf minerals contents of rice plants [Aluminum (Al), Boron (B), Cadmium (Cd), Chromium (Cr), Copper (Cu), Iron (Fe), Manganese (Mn), Molybdenum (Mo), Nickel (Ni), Lead (Pb), Rubidium (Rb), Selenium (Se), Strontium (Sr), Titanium (Ti), Thallium (Tl), Zinc (Zn)] grown under irradiances of 150 µmol m^{-2} s^{-1} (depicted by white bars) and 300 µmol m^{-2} s^{-1} (represented by gray bars), and cultivated in either ambient (ACO_2) or elevated (ECO_2) CO_2 conditions. The values presented are means ± SD derived from 3 replicates.

Supplemental Figure S3

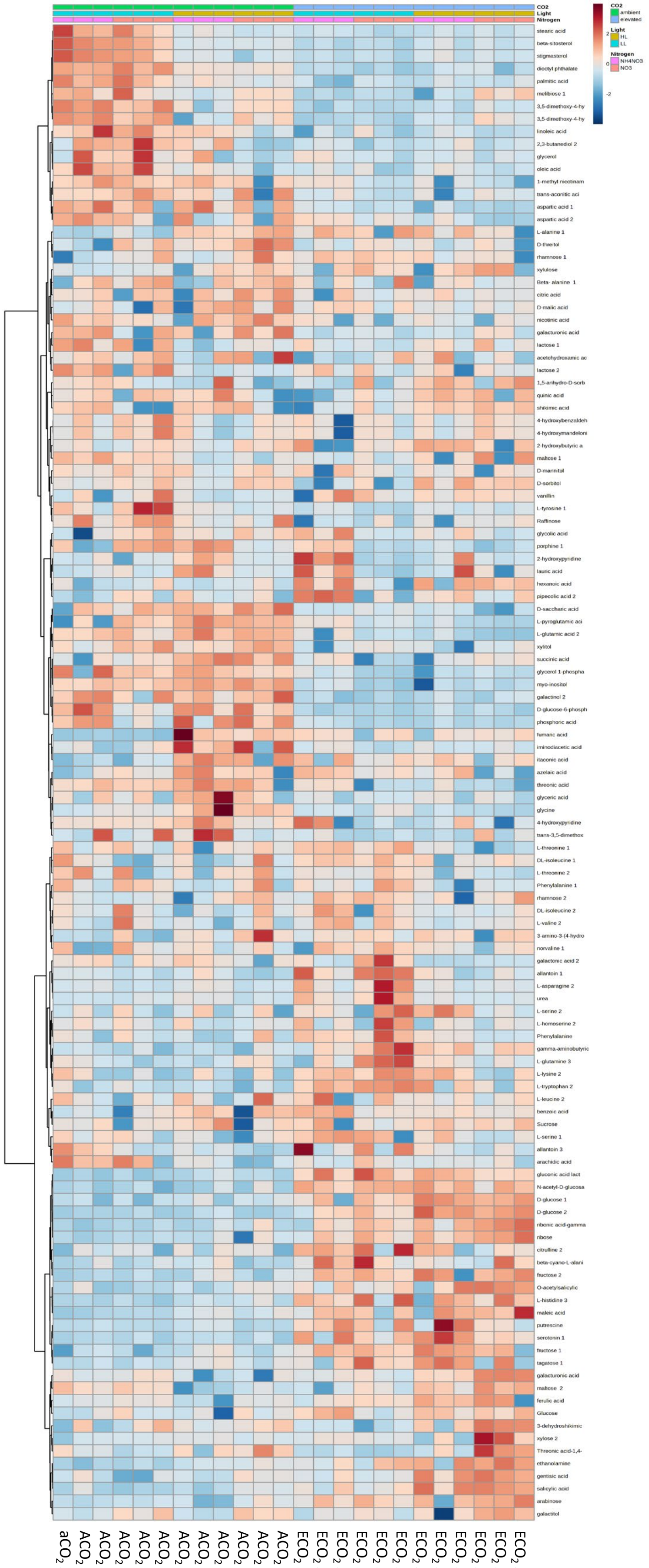

Fig. S3 illustrates the impact of various N forms (NO_3 or NH_4NO_3) on leaf metabolites contents of rice plants grown under irradiances of 150 µmol m^{-2} s^{-1} (depicted by white bars) and 300 µmol m^{-2} s^{-1} (represented by gray bars), and cultivated in either ambient (ACO_2) or elevated (ECO_2) CO_2 conditions. The values presented are means ± SD derived from 3 replicates.

Supplemental Figure S4

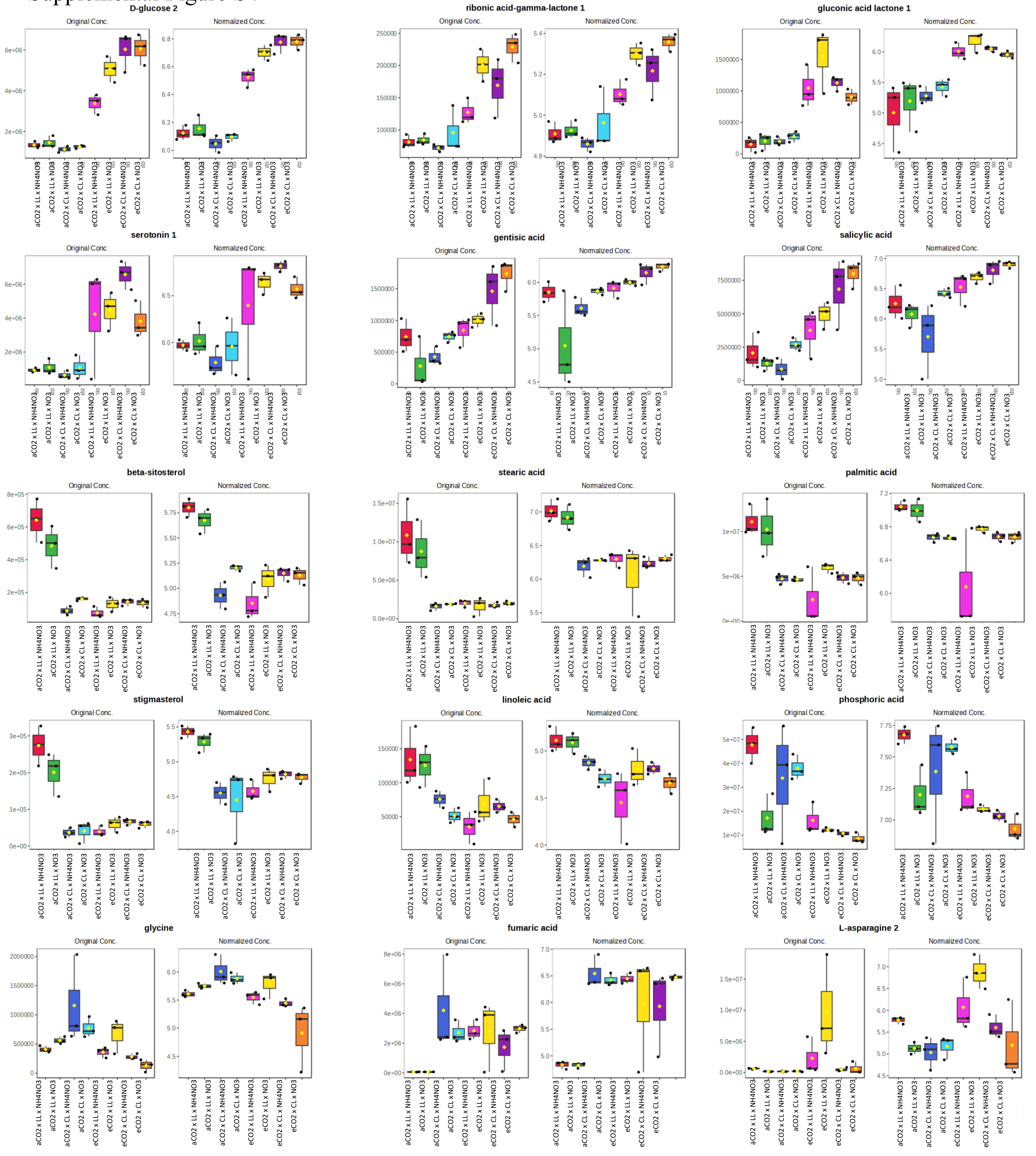

Fig. S4 illustrates the impact of various N forms (NO_3 or NH_4NO_3) on leaf metabolites contents of rice plants grown under irradiances of 150 µmol m^{-2} s^{-1} (depicted by white bars) and 300 µmol m^{-2} s^{-1} (represented by gray bars), and cultivated in either ambient (ACO_2) or elevated (ECO_2) CO_2 conditions. The values presented are means ± SD derived from 3 replicates.

Supplemental Figure S5

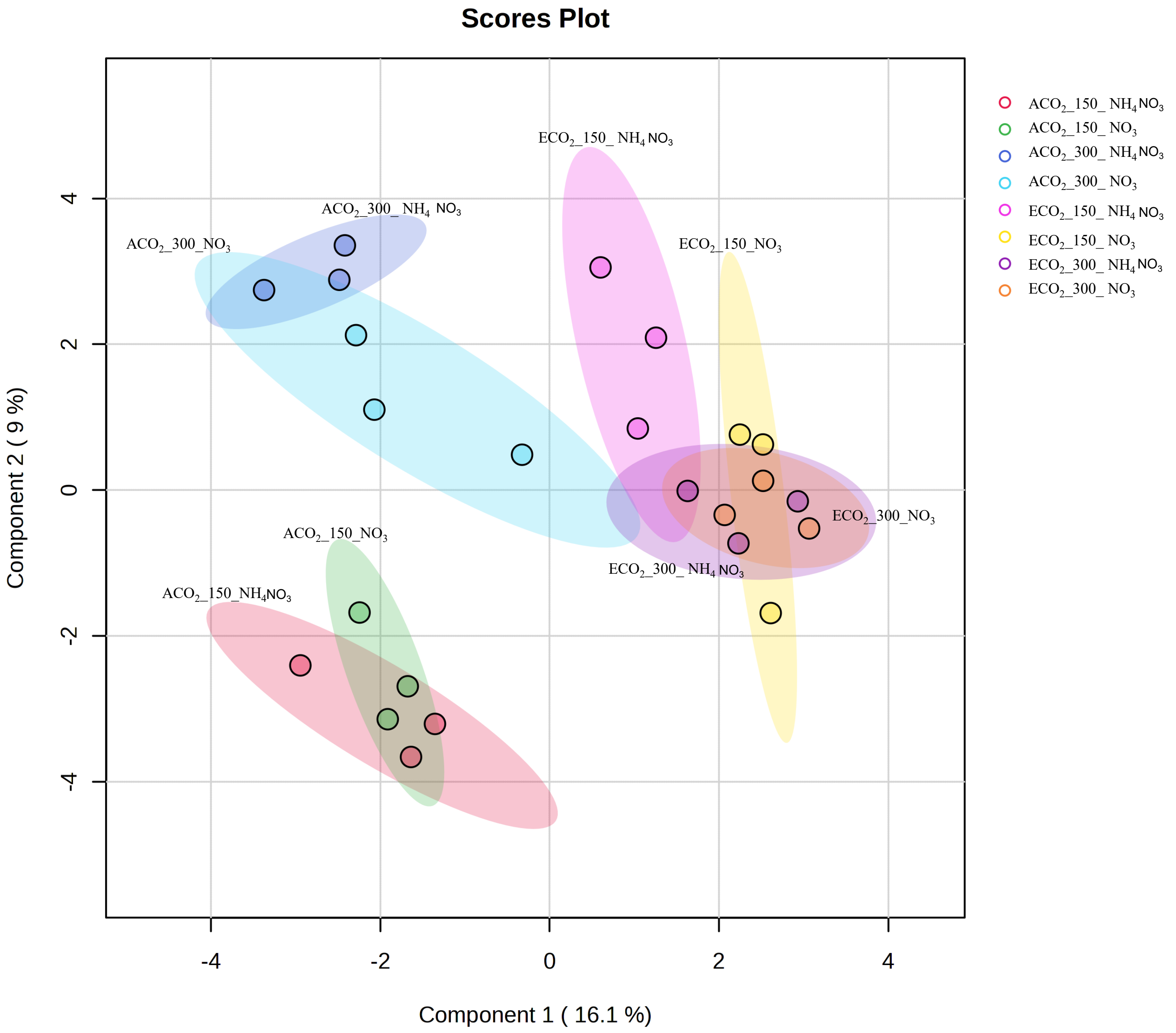

Fig. S5 illustrates the impact of various N forms (NO_3 or NH_4NO_3) on different samples of the metabolome of rice plants grown under irradiances of 150 µmol m^{-2} s^{-1} (depicted by white bars) and 300 µmol m^{-2} s^{-1} (represented by gray bars), and cultivated in either ambient (ACO_2) or elevated (ECO_2) CO_2 conditions represented into a PCA plot. The values presented are means ± SD derived from 3 replicates.

Supplemental Figure S6

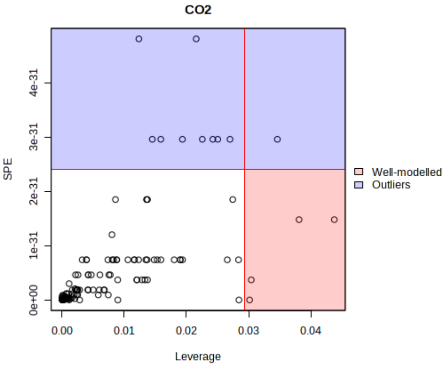

N-acetyl-D-glucosamine 1	0.0437771	1.47911E-31
gluconic acid lactone 1	0.0381051	1.47911E-31
gamma-aminobutyric acid (GABA)	0.0304216	3.69779E-32
D-glucose 2	0.030162	0.0

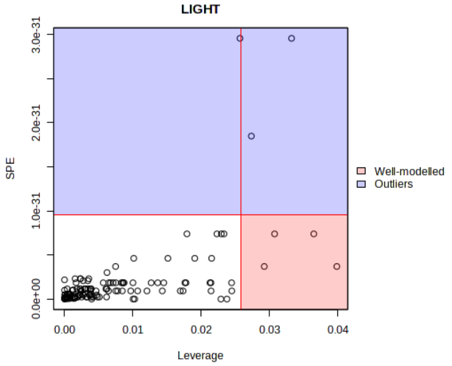

xylose 2	0.0398388	3.69779E-32
arabinose	0.0364709	7.39557E-32
Beta- alanine 1	0.0307343	7.39557E-32

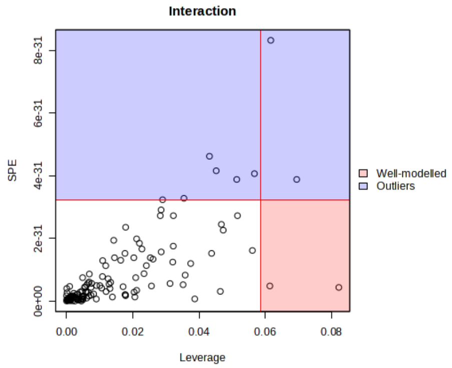

L-asparagine 2	0.0822069	4.39112E-32
salicylic acid	0.0613879	4.85334E-32

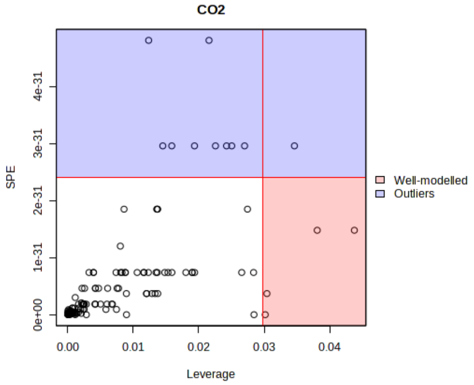

N-acetyl-D-glucosamine 1	0.0437771	1.47911E-31
gluconic acid lactone 1	0.0381051	1.47911E-31
gamma-aminobutyric acid (GABA)	0.0304216	3.69779E-32
D-glucose 2	0.030162	0.0

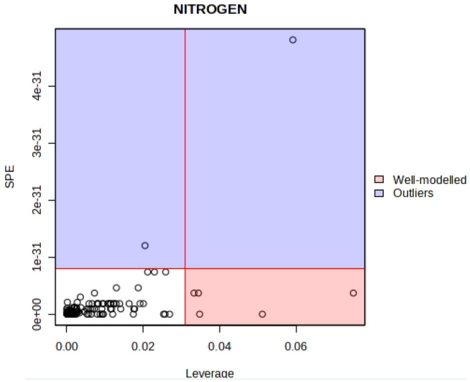

fumaric acid	0.0750022	3.69779E-32
allantoin 1	0.0511862	0.0
gluconic acid 2	0.0347667	0.0
arachidic acid	0.0344491	3.69779E-32
allantoin 3	0.0333046	3.69779E-32

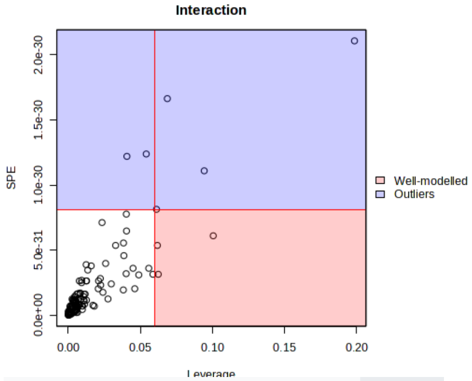

galacturonic acid 1	0.100572	6.10135E-31
glycine	0.0624402	3.14312E-31
beta-sitosterol	0.0618336	5.36179E-31

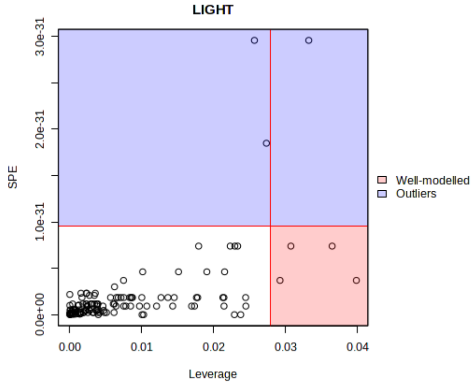

xylose 2	0.0398388	3.69779E-32
arabinose	0.0364709	7.39557E-32
Beta- alanine 1	0.0307343	7.39557E-32
ethanolamine	0.029226	3.69779E-32

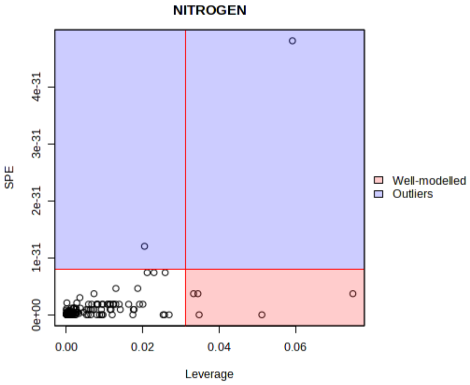

fumaric acid	0.0750022	3.69779E-32
allantoin 1	0.0511862	0.0
gluconic acid 2	0.0347667	0.0
arachidic acid	0.0344491	3.69779E-32
allantoin 3	0.0333046	3.69779E-32

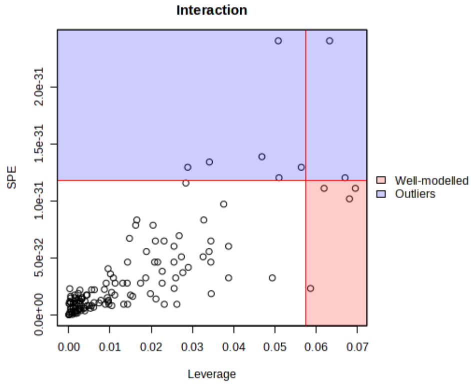

Threonic acid-1,4-lactone B	0.0694917	1.10934E-31
2,3-butanediol 1	0.0680554	1.01689E-31
porphine 1	0.061912	1.10934E-31
2,3-butanediol 2	0.0586214	2.31112E-32

Fig. S6 illustrates the impact of various N forms (NO_3 or NH_4NO_3) on leaf metabolites contents of rice plants grown under irradiances of 150 µmol m^{-2} s^{-1} (depicted by white bars) and 300 µmol m^{-2} s^{-1} (represented by gray bars), and cultivated in either ambient (ACO_2) or elevated (ECO_2) CO_2 conditions represented using a leverage plots. The values presented are means ± SD derived from 3 replicates.
